# Efficacy of Ciprofloxacin/Celecoxib combination in zebrafish models of amyotrophic lateral sclerosis

**DOI:** 10.1002/acn3.51174

**Published:** 2020-09-11

**Authors:** Hagit Goldshtein, Alexandre Muhire, Virginie Petel Légaré, Avital Pushett, Ron Rotkopf, Jeremy M. Shefner, Randall T Peterson, Gary A. B. Armstrong, Niva Russek‐ Blum

**Affiliations:** ^1^ The Dead Sea Arava Science Center Auspices of Ben Gurion University Central Arava 86815 Israel; ^2^ Department of Neurology and Neurosurgery Montreal Neurological Institute Faculty of Medicine McGill University Montreal QC H3A 0G4 Canada; ^3^ NeuroSense Therapeutics Ltd Medinat Hayehudim 85 Herzeliya 4676670 Israel; ^4^ Bioinformatics and Biological Computing Unit Life Sciences Core Facilities Weizmann Institute of Science Rehovot 7610001 Israel; ^5^ Barrow Neurological Institute, University of Arizona College of Medicine Phoenix, Creighton University College of Medicine Phoenix Phoenix AZ 85013 USA; ^6^ College of Pharmacy University of Utah Salt Lake City UT 84112 USA

## Abstract

**Objective:**

To evaluate the efficacy of a fixed‐dose combination of two approved drugs, Ciprofloxacin and Celecoxib, as a potential therapeutic treatment for amyotrophic lateral sclerosis (ALS).

**Methods:**

Toxicity and efficacy of Ciprofloxacin and Celecoxib were tested, each alone and in distinct ratio combinations in SOD1 G93R transgenic zebrafish model for ALS. Quantification of swimming measures following stimuli, measurements of axonal projections from the spinal cord, neuromuscular junction structure and morphometric analysis of microglia cells were performed in the combination‐ treated *vs* nontreated mutant larvae. Additionally, quantifications of touch‐evoked locomotor escape response were conducted in treated *vs* nontreated zebrafish expressing the *TARDBP* G348C ALS variant.

**Results:**

When administered individually, Ciprofloxacin had a mild effect and Celecoxib had no therapeutic effect. However, combined Ciprofloxacin and Celecoxib (Cipro/Celecox) treatment caused a significant increase of ~ 84% in the distance the SOD1 G93R transgenic larvae swam. Additionally, Cipro/Celecox elicited recovery of impaired motor neurons morphology and abnormal neuromuscular junction structure and preserved the ramified morphology of microglia cells in the SOD1 mutants. Furthermore, larvae expressing the TDP‐43 mutation displayed evoked touch responses that were significantly longer in swim distance (110% increase) and significantly higher in maximal swim velocity (~44% increase) when treated with Cipro/Celecox combination.

**Interpretation:**

Cipro/Celecox combination improved locomotor and cellular deficits of ALS zebrafish models. These results identify this novel combination as effective, and may prove promising for the treatment of ALS.

## Introduction

ALS is a fatal neurodegenerative disease which affects motor neurons (MNs) in the brain and spinal cord, leading to muscle paralysis and eventually death within 3–5 years from symptoms onset^1^, ^2^. Diverse pathophysiological mechanisms are associated with ALS, including deposition of RNA and protein aggregates, aberrations in RNA regulation, defects in axonal transport and neuronal excitability.^1^, ^2^ In parallel, neuroinflammation is believed to play a role in the disease process.^3^ Despite considerable advances made in understanding the mechanisms underlying ALS, treatment possibilities remain limited.^4^ Due to the complex range of cellular events that lead to ALS, a combined treatment of multiple downstream pathways may be a beneficial therapeutic strategy.^5^

Increasing evidence suggests that RNA metabolism, including microRNAs (miRNAs) may play an important role in the pathomechanism of ALS. Dysregulated microRNAs are found in microglia cells, MNs, skeletal muscles of ALS patients and animal models^6^, ^7^, ^8^ and in human ALS‐induced pluripotent stem cells.^9^ Ciprofloxacin is an approved synthetic antibiotic compound belonging to the fluoroquinolone family^10^ that was found to possess a substantial RNA interference (RNAi)‐enhancing activity.^11^

Increased cyclooxygenase‐2 (COX2) expression, and therefore prostaglandin E2 (PGE2) levels, have been reported in ALS animal models and patients.^12^, ^13^, ^14^ Celecoxib, an approved nonsteroidal antiinflammatory drug (NSAID) specifically inhibits COX2,^15^ potentially interfering with glutamate‐induced excitotoxicity,^16^ inflammation^17^ and oxidative stress (OS)‐related toxicity,^18^ processes thought to play a major role in neuronal damage in ALS. Although Celecoxib prolongs survival of the superoxide dismutase 1 (SOD1) G93A mouse model,^19^ treatment of ALS patients with high Celecoxib dosing did not result in a beneficial clinical outcome or delayed disease progression.^20^

However, combined activity of Ciprofloxacin and Celecoxib has been demonstrated, where low doses of Celecoxib played a synergistic role with Ciprofloxacin in regulating OS and inflammation.^21^ Furthermore, Celecoxib has been shown to possess the ability to inhibit multidrug resistance (MDR1) efflux pumps, thus leading to the accumulation of Ciprofloxacin inside cells.^22^, ^23^

Based on the data suggesting potential synergism, and the rationale to target multiple downstream pathways, we conducted a Ciprofloxacin and Celecoxib combination treatment in two well‐characterized genetic ALS zebrafish models carrying the *SOD1* or TAR DNA binding protein 43 (*TARDBP*) mutations. Both these models show the hallmarks of ALS, including defective motor performance, irregular MNs, loss of neuromuscular connectivity, and muscle atrophy.^24^, ^25^, ^26^, ^27^ The zebrafish model was chosen for this purpose due to its well‐established locomotor phenotypes and high amiability to genetic manipulations which allow high‐throughput drug screening.^28^ In addition, larval zebrafish MNs and neuromuscular junctions (NMJs) are functionally and anatomically similar to those of humans, as well as other cell types and molecular pathways in the nervous and immune systems.^29^ Furthermore, a growing number of potential therapies discovered in zebrafish models have reached clinical trials phases.^30^, ^31^, ^32^

Here we show that Ciprofloxacin elicited a significant and dose‐dependent improvement in locomotor activity of the SOD1 zebrafish model. Combined with a very low dose of Celecoxib, this result was greatly enhanced. Similar results were obtained using the *TARDBP* zebrafish model. Furthermore, Cipro/Celecox caused a significant recovery of the impaired MN axonopathy deficits and NMJ abnormalities in SOD1 mutants and finally, morphometric analysis of microglia showed that the combined treatment preserved the ramified morphology of these cells. By applying locomotor and morphometric characterizations, we defined a specific fixed‐dose combination of Cipro/Celecox that showed beneficial effects in ALS zebrafish models.

## Methods

### Zebrafish

Adult and larval zebrafish (*Danio rerio*) were bred and reared at 28.5 °C under 12 h/12 h light/dark cycle, according to standard protocols.^33^ All experiments using Tg(SOD1:SOD1G93R) zebrafish were approved by the Ben Gurion University Committee of Use and Care of Animals and conducted at the ADSSC institute, Israel. All *TARDBP* zebrafish experiments were approved by the Canadian Council for Animal Care and conducted at the Montreal Neurological Institute at McGill University, Montreal, Canada. Tg(SOD1:SOD1G93R) line was kindly provided by Prof. Christine E. Beattie^24^ and the apolipoprotein‐E Tg (Apo‐E:GFP) line was kindly provided by Prof. Francesca Peri.^34^

### Drug administration protocol

Mutant SOD1 (mSOD1) or wild type (WT) fish were treated with distinct concentrations of Celecoxib (Prudence Pharma Chem, India), Ciprofloxacin (Neuland Laboratory LTD, India) or Riluzole (R116, Sigma‐Aldrich) with 0.1% dimethyl sulfoxide (DMSO) as background. DMSO drives swimming effect in behavioral toxicity bioassays.^35^ Therefore, each individual experiment includes its own control containing 0.1% DMSO. Celecoxib was dissolved to a stock solution of 10mM in 100% DMSO. Ciprofloxacin was dissolved to a stock solution of 100 mM in ddH2O. Compounds were diluted in zebrafish raising buffer^33^ and their pH was adjusted to 6.7 using NaOH. Larvae were treated at 3 days postfertilization (dpf) and the solutions were replaced by fresh ones at 5 dpf. *TARDBP*‐injected or WT embryos were dechorionated and placed in culture flasks containing final drug concentrations of Celecoxib (PZ0008, Sigma‐Aldrich), Ciprofloxacin (PHR1044, Sigma‐Aldrich**)** or their combination at exactly 34 hours post fertilization (hpf). Embryos were treated for 18 hours and then placed in a drug‐free raising buffer prior to evaluating touch‐evoked responses.

### Toxicity evaluation

Following treatment, fish were observed to evaluate toxicity. Acute toxicity such as apoptosis/necrosis, specific organ toxicity (liver, kidney, head, eyes, etc.), cardiovascular system abnormalities (heart rate, morphology, hemorrhage and edema) and behavioral toxicity were recorded according to accepted procedures.^36^

### Motor performance of SOD1 G93R larvae

The DanioVision tracking system (Ethovision XT 13.0; Noldus Information Technology, the Netherlands), was used for swimming measurements. Each animal was tested for its x,y position using dynamic subtraction taking 30 frames per second. Larvae were evaluated for locomotor activity at 6 dpf. Individual larvae were placed in 48‐well plates, which were put in the DanioVision system with light on for 20 min prior to the beginning of the trial. Larvae were subjected to 10 min dark followed by 10 min light. Larval activity was measured and analyzed during the last 10‐min light period, measuring recovery from dark/ light transition. Each plate tested contained control animals from the same spawn. Each experiment was repeated using distinct spawns. Experiments were conducted at the same time of day (10:00–14:00) at 24–25°C. Activity parameters were extracted to Excel and analyzed using Access and R.

### Whole mount immunofluorescent staining

Larvae were fixed in 4% paraformaldehyde/phosphate‐buffered saline (PBS), washed in PBS, dehydrated using methanol 100% and stored at −20°C overnight, then treated with 100% acetone in −20°C for 10 minutes. The rehydrated samples were blocked in blocking solution (PBS + 10% goat serum + 1% DMSO + 0.3% Triton X100) that was then replaced with fresh blocking solution with commercial primary antibodies. Acetylated tubulin antibodies (T6793; Sigma‐Aldrich; 1:1000) were used followed by Alexa Fluor 633 goat anti‐ mouse antibodies (A‐21052; Life Technologies; 1:200). For BTX + SV2 staining, samples were incubated with Alexa Fluor 488 conjugated α‐Bungarotoxin (B13422; Invitrogen; 1:300) overnight at 4°C, washed, incubated overnight at 4°C with synaptic vesicle antibodies (AB_2315387; DSHB; 1:100), washed and incubated overnight at 4°C with Alexa Fluor 633 goat anti‐mouse antibodies.

### Image acquisition and analysis

Fluorescent images were obtained using Plan‐Neofluar 40x/0.75 objective on a Zeiss AxioImagerM2 microscope with ApoTome for optical sectioning (Zeiss, Germany). Images of MNs and microglia were analyzed using Imaris 9.0.0 (Bitplane, Switzerland). Filament tracer (Bitplane), which automatically detects filament‐like structures, was used to outline and quantify the axons and microglia processes. Images of NMJs were analyzed using the open source FIJI image‐processing package, NMJ puncta were counted using the Cell Counter plugin.

### Preparation and injection of *TARDBP* mRNA

Human *TARDBP* cDNA was obtained from Open Biosystems. The mutation encoding the G348C variant was introduced using site‐directed mutagenesis in the appropriate vector using QuikChange XL Site‐Directed Mutagenesis Kit (Stratagene).^25^, ^37^ mRNA preparations and injections into 1 cell stage zygotes were performed as described.^25^

### Touch‐evoked locomotor behavior

Assessment of zebrafish locomotor patterns was performed at room temperature (24–25°C) in larvae aged 54–56 hpf. Larvae were placed in the middle of a circular arena (150 mm diameter) filled with drug‐free raising buffer. Burst swimming was initiated by a single touch to the tail and locomotor activity was recorded from above (sampled at 30 Hz; Grasshopper 2 camera, Point Grey Research). Swim distance and maximum swim velocity were quantified using the manual tracking plugin for ImageJ.

### Statistics

Total swimming distances (calculated in 1 minute time bins) were averaged and compared between treatments using a linear mixed effects model, with treatment as a fixed effect and a random intercept for each plate. A Tukey post hoc test was used to compare all treatments. Additionally, all distances (averaged per fish) were scaled per plate divided by the control mean. These scaled distances were compared between treatments by a one‐way ANOVA, followed by a Tukey post hoc test. Statistics were conducted using the R, v.3.6 software.

For touch‐evoked locomotor experiments, SigmaPlot 12.0 integrated with SigmaStat was used to assess data groupings for significance. Statistical analyses used one‐way ANOVA, followed by a post hoc Holm‐Sidak multiple comparison test or a Kruskal‐Wallis one‐way ANOVA on ranks followed by a post hoc Dunn’s multiple comparison test.

## Result

### Cipro/Celecox treatment improved motor performance of SOD1 G93R larvae

**Figure 1:**
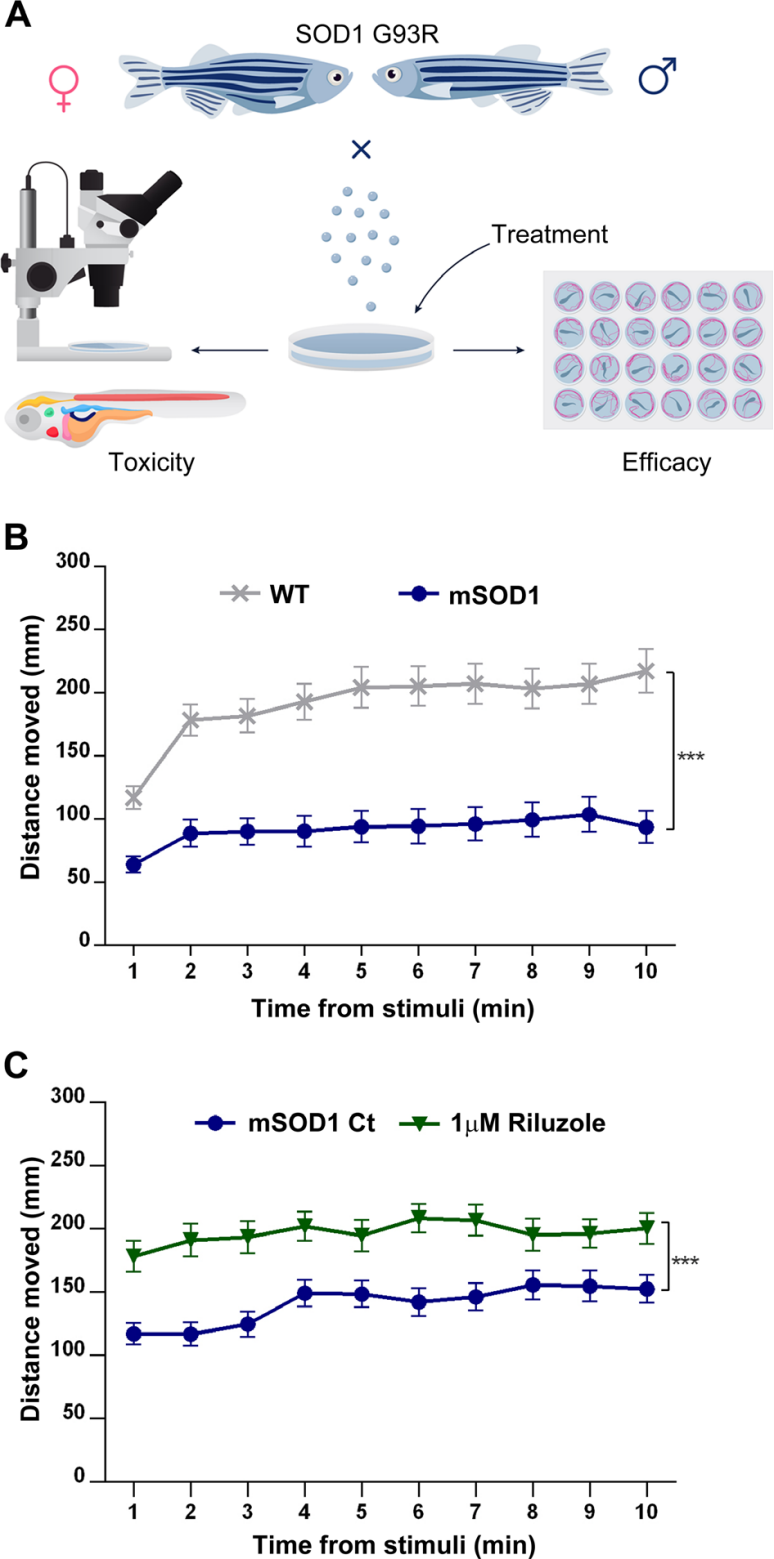
Evaluation of drug toxicity and efficacy in SOD1 G93R mutant fish. (A) Scheme describing the procedure undertaken to evaluate drug toxicity and efficacy in mSOD1 larvae. Toxicity evaluation included acute toxicity, such as apoptosis/necrosis, specific organ toxicity (liver, kidney, heart, etc), cardiovascular system abnormalities (heart rate, morphology, hemorrhage and edema) and behavioral toxicity. Efficacy was evaluated by analyzing locomotor ability and endurance. (B) 6 dpf WT and mSOD1 larvae were subjected to dark/ light transition. The distance they swam after light stimuli per 1 min time bin was measured and averaged. (***P < 0.001; linear mixed model; n = 72 per each group). (C) mSOD1 larvae were treated with vehicle (0.1% DMSO; Ct) or 1 µmol/L Riluzole. The distance they swam per 1 min time bin after light stimuli was measured and averaged. (***P < 0.001; linear mixed model; *n* = 72).

The SOD1 G93R mutant zebrafish (referred to herein as mSOD1) ALS model, which shows motor behavioral and axonal phenotypes similar to MN disease^24^, was used in our drug screening platform to evaluate the toxicity and efficacy of two approved drugs‐ Ciprofloxacin and Celecoxib, individually and in combination (Fig. 1A). To quantify the motor ability of 6 dpf larvae, their swimming distance was measured following dark/ light transition for 10 min by use of automated high‐throughput tracking analysis. Analysis of the averaged distance that the larvae moved per time bin of 1 min following light stimuli revealed that mSOD1 larvae swam significantly shorter distances compared to their WT counterparts (*P* < 0.001; Fig. 1B).

Following the external light stimuli, the activity of both mSOD1 and WT larvae decreased precipitously, resulting in sudden freezing. This freezing was followed by a gradual increase to a maximum sustained level after approximately 7–10 min in light. The mSOD1 locomotor activity was greatly diminished compared to WT following external light stimuli and this part was chosen for further screening analysis as the most indicative and relevant (Fig. S1).

Following initial calibration, the ability of Riluzole, one of two drugs approved to slow disease progression in ALS^4^, was tested to modify locomotor activity of mSOD1 larvae. Treatment with Riluzole caused a significant increase in locomotor activity of 6 dpf mSOD1 larvae (*P* < 0.001; Fig. 1C).

**Figure 2:**
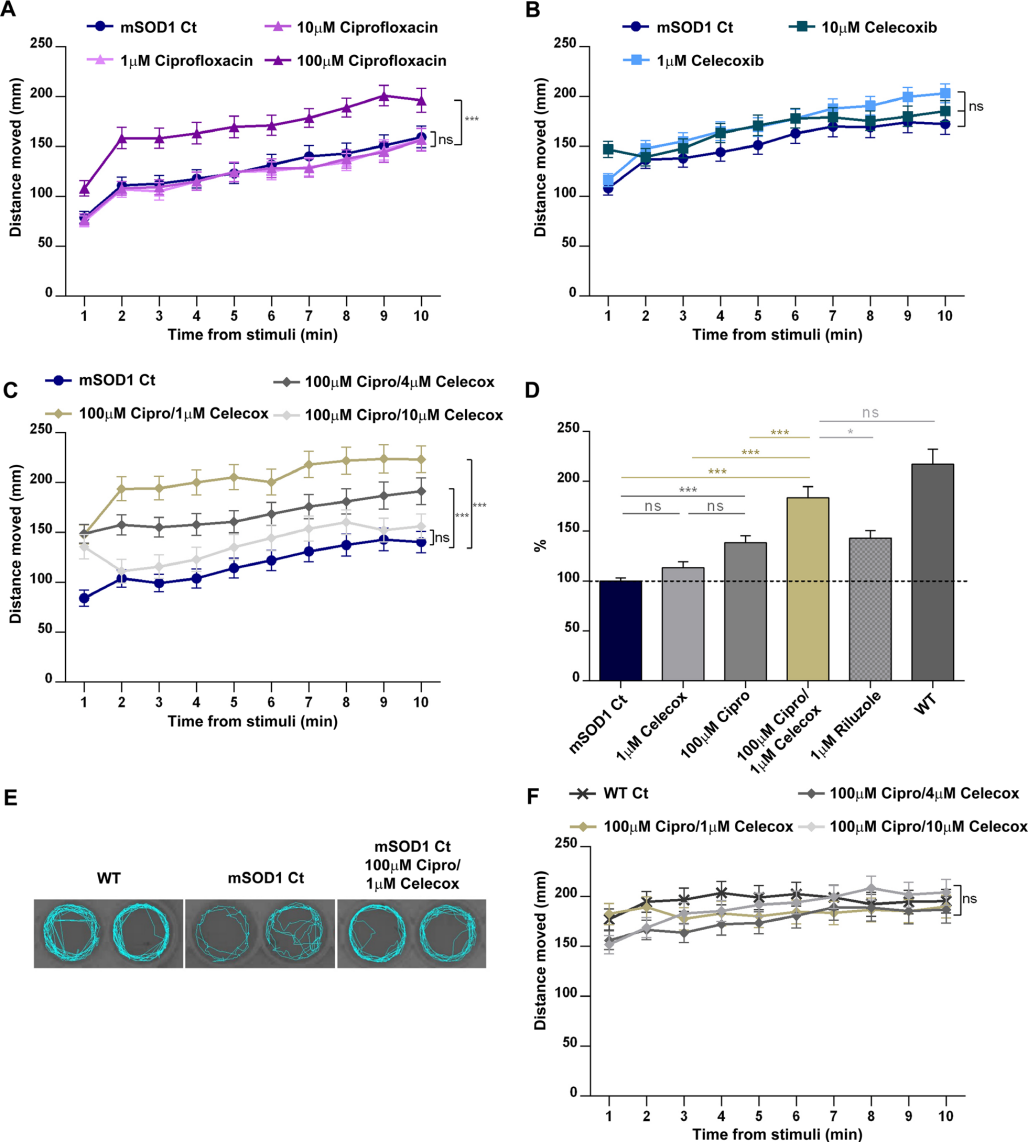
Cipro/Celecox treatment specifically improved motor performance of mSOD1 larvae. mSOD1 larvae were treated with (A) vehicle (0.1% DMSO; Ct), 1 µmol/L, 10 µmol/L or 100 µmol/L Ciprofloxacin; (B) 1 µmol/L or 10 µmol/L Celecoxib; or (C) distinct combination ratios of the drugs, and were then subjected to dark/ light transition protocol, and the distance they swam per 1 min time bin following light stimuli was measured and averaged. (ns = non significant; ***P < 0.001; linear mixed model, Tukey post hoc test, *n* = 96 for each treatment group in a, *n* = 117 in b, *n* = 96 in c). (D) The increase (%) in the distance the mSOD1‐treated larvae swam compared to control mSOD1. All distances swam were averaged per fish for the whole period following light stimuli, scaled per plate and compared between treatments (ns = non significant; *P < 0.05; ***P < 0.001; one‐way ANOVA, Tukey post hoc test). (E) Representative swimming tracks of individual larvae for 60 s. Recovery of swimming pattern was observed following treatment with Cipro/Celecox. (F) WT larvae were treated with vehicle (0.1% DMSO; Ct), or distinct combination ratios of the drugs, and the distance they swam after stimuli per time bin of 1 min was measured and averaged. (ns = non significant; linear mixed model, Tukey post hoc test, n = 96 for each treatment group).

Ciprofloxacin was first evaluated, using screening concentrations ranging between 1 and 100 µmol/L. This dose range generally identifies the highest concentrations that can lead to nonspecific toxicity and death, and is low enough to identify weakly active compounds^28^. Ciprofloxacin was introduced into the raising buffer of the mSOD1 larvae at day 3 and 5 in three final concentrations: 1 µmol/L, 10 µmol/L and 100 µmol/L. In all Ciprofloxacin doses, drug‐induced behavioral, morphological toxicity or mortality were not observed (data not shown). The two low doses did not have an effect on locomotor ability, while the 100 µmol/L Ciprofloxacin treatment significantly improved locomotion in mSOD1 larvae (*P* < 0.001; Fig. 2A). Increase in Ciprofloxacin dosing (up to 500µM) did not cause further improvement or toxicity (Fig. S2).

Celecoxib was then introduced to the raising buffer of mSOD1 larvae in distinct concentrations to evaluate toxicity and efficacy. Toxicity was evident at 30 µmol/L Celecoxib treatment: Following the first treatment, pericardial edema and decreased heart rate were evident in all larvae treated, and all larvae died following the second treatment. mSOD1 larvae treated with 10 µmol/L or lower Celecoxib concentrations exhibited no obvious drug‐induced effects on gross morphology or mortality. Analysis of the averaged distance that treated larvae moved per 1 minute time bin following stimuli, showed that treatment with 1 or 10 µmol/L Celecoxib by itself, did not induce a substantial effect on locomotor ability of mSOD1 larvae (Fig. 2B). Further reduction in Celecoxib doses had no effect on mSOD1 activity (Fig. S3).

Despite Celecoxib’s lack of efficacy, combining it with Ciprofloxacin was of interest, as they have previously been shown to synergize in regulating ALS‐relevant pathways^21^. The distinct ratios between the two drugs were used to evaluate their potential synergistic effect on the locomotor activity of mSOD1 larvae. Celecoxib, 1, 4 and 10 µmol/L, in combination with the most potent concentration of Ciprofloxacin (100 µmol/L) was tested. No cardiovascular abnormalities (i.e., heart rate, morphology, hemorrhage and edema) or mortality were observed in all combinations used (100 µmol/L Cipro/ 1 µmol/L Celecox; 100 µmol/L Cipro/ 4 µmol/L Celecox; 100 µmol/L Cipro/ 10 µmol/L Celecox). The 100 µmol/L Ciprofloxacin and 1 µmol/L Celecoxib combination caused the most robust improvement in mSOD1 locomotor ability (*P* < 0.001; Fig. 2C) and was therefore used in the following experiments.

Comparison of the effects of the various treatments on mSOD1 G93R fish average motor activity, demonstrated that 100µM Ciprofloxacin induced a significant increase in swimming distance (38.5%; *P* < 0.001; Fig. 2D), which was similar to that of Riluzole (43.2%; *P* < 0.001) and Celecoxib at 1 µmol/L had no significant effect. Strikingly, when utilizing both compounds (100 µmol/L Cipro/ 1 µmol/L Celecox), a significant increase of 83.5% in locomotor activity of mSOD1 larvae was observed, suggesting a synergistic effect between the two drugs (*P* < 0.001; Fig. 2C‐E).

During the dark period, when dark enhanced activity is demonstrated, the activity differences between the WT and mSOD1 larvae are milder than following external light stimuli (Fig. S1). Cipro/Celecox caused a significant increase in locomotor activity of treated mSOD1 larvae also during the dark phase, providing the same support for combining the drugs, as in the light condition (Table S1).

In order to verify that the locomotor activity enhancement was specific to mSOD1 larvae, WT larvae were treated with distinct Cipro/Celecox fixed‐dose combination ratios. The Cipro/Celecox combinations did not have an effect on WT larvae swimming distance (Fig. 2F), suggesting that the Cipro/Celecox combination enhances locomotor ability specifically by targeting mechanisms underlying SOD1 ALS.

### Cipro/Celecox treatment fully recovered mSOD1 axonopathy

**Figure 3:**
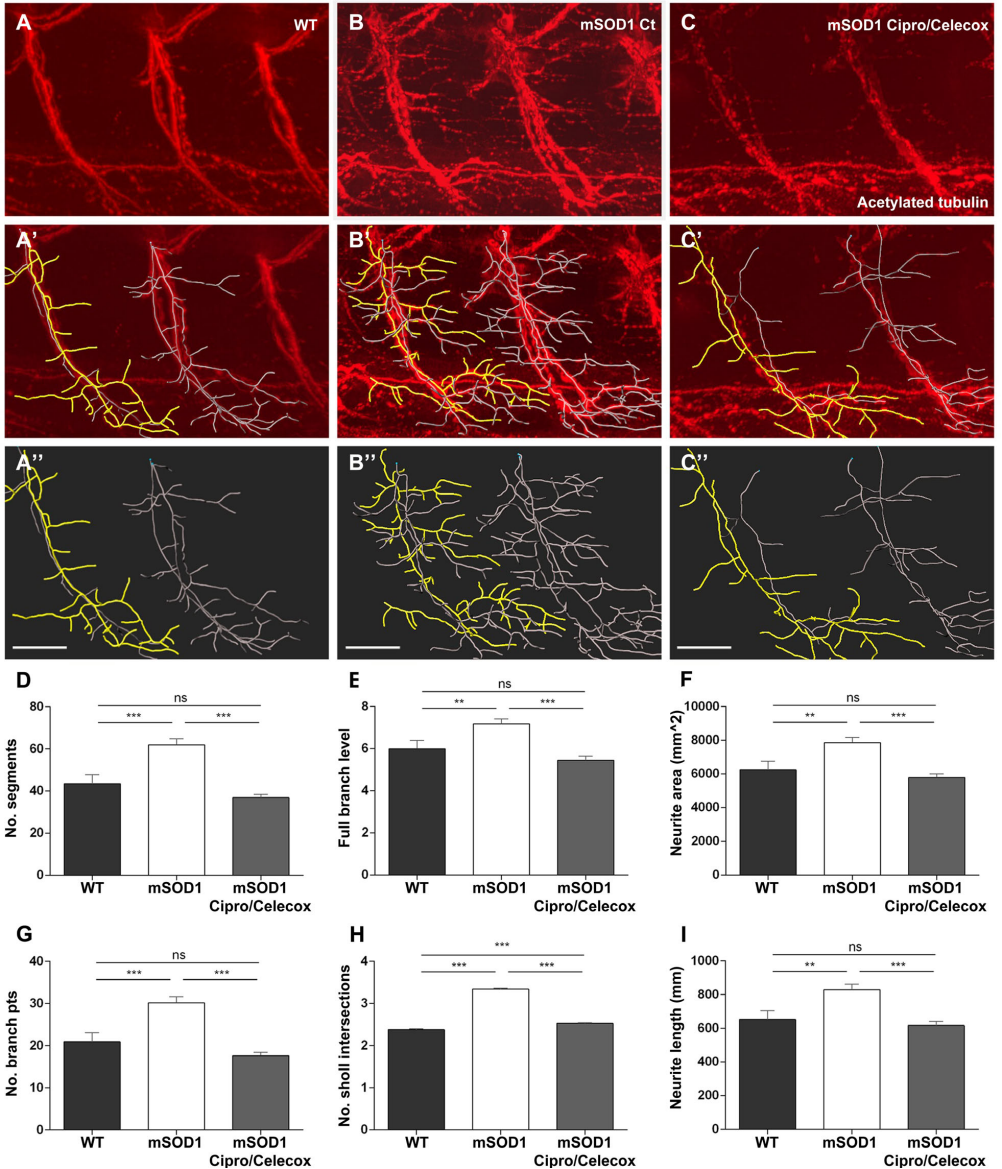
Cipro/Celecox treatment fully recovered mSOD1 axonopathy. (A‐C) 3D reconstructed apotome z‐stack images of branching motor neurons in the trunk of 6dpf larvae immunostained with antiacetylated tubulin antibodies (segments S10‐ S12). (A'‐C'') The backbone (colored processes) of the motor neurons traced with the Filaments analysis of Imaris software. In yellow‐ single motor neuron. WT (A–A''), control mSOD1 (B‐B'') and 100 µmol/L Cipro/ 1 µmol/L Celecox‐ treated mSOD1 larvae (C–C''). (scale bars = 40 µm). (D–I) Morphometric analysis measurements of individual motor neuron in WT, mSOD1 control and mSOD1 treated with 100 µmol/L Cipro/ 1 µmol/L Celecox. Graphs indicate (D) number of segments, (E) full branch level, (F) neurite area, (G) number of branch points, (H) number of Sholl's intersections and (I) neurite length. (ns = non significant; **P < 0.01; ***P < 0.001; one‐way ANOVA, Tukey post hoc test, *n* = 15 for control and treated mSOD1 fish and *n* = 11 for WT fish).

In light of our findings that Cipro/Celecox treatment improved motor functions of mSOD1 larvae we next aimed to test whether this improvement is accompanied by neuromorphological changes. Spinal MN axonal projections (segments 10–12) of 6dpf treated and nontreated mSOD1 larvae were stained for acetylated tubulin and analyzed for axonal morphometry compared to WT parameters (Fig. 3).

WT larvae predominantly exhibited normal MN morphometry, with long and moderately branched axons (Fig. 3A‐A''). On the contrary, the mSOD1 control group exhibited phenotypes consisting of disorganized, excessively branched motor neuronal axons (Fig. 3B‐B'').

mSOD1 larvae treated with 100 µmol/L Cipro/ 1 µmol/L Celecox combination, showed a significant recovery of the mutant morphology, and with nearly normal axon morphology (Fig. 3C‐C''). All parameters of MN morphology, including number of branches, branching level, neurite area, branching points, neurite length and their spreading in Sholl analysis, showed a significant reduction in axonopathy in Cipro/Celecox‐treated compared to nontreated mSOD1 larvae, similar to WT axon morphology (*P* < 0.001; Fig. 3D–I).

### Cipro/Celecox rescued orphaned pre‐ and post‐synaptic components in mSOD1 larvae

**Figure 4:**
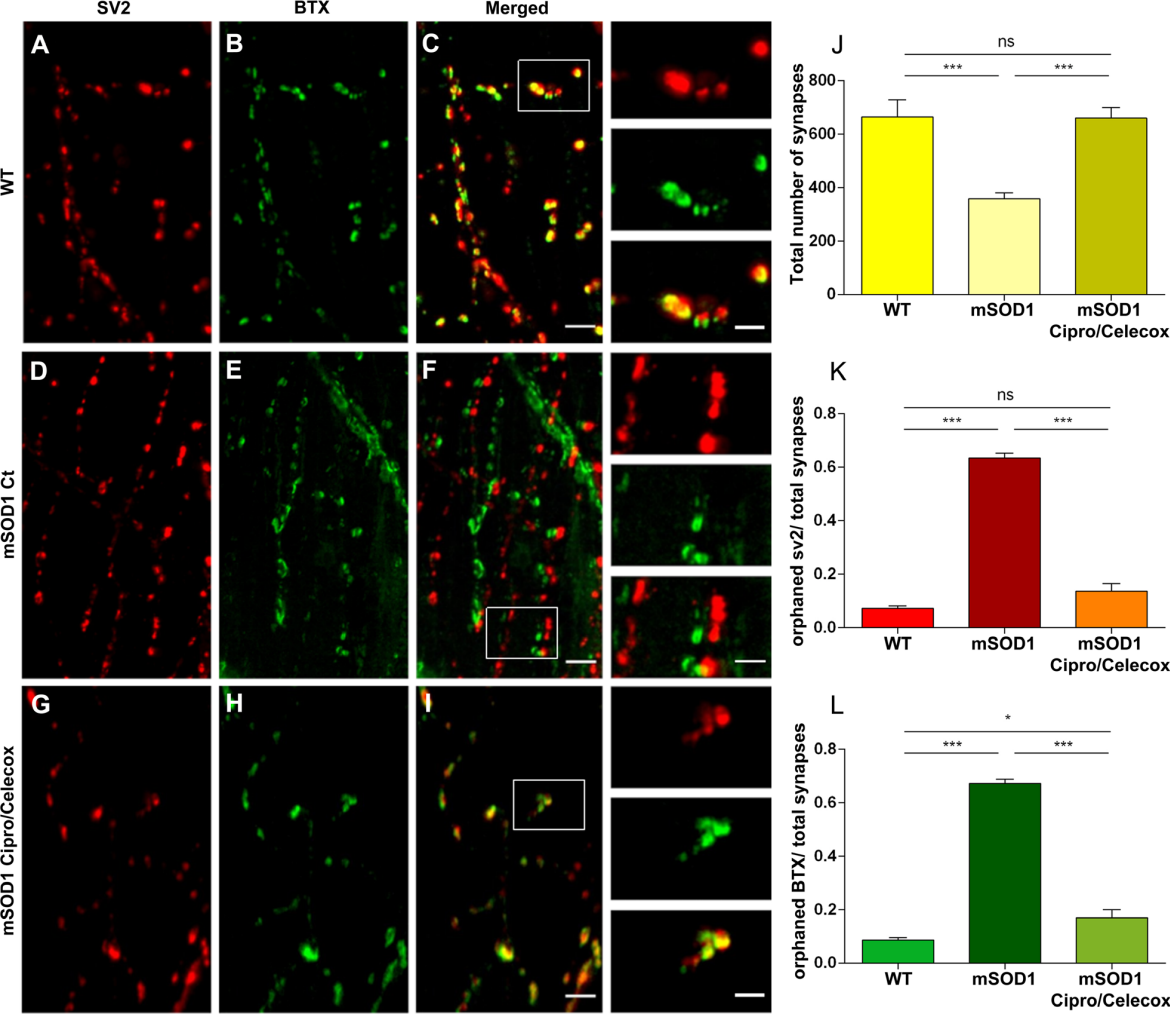
Cipro/Celecox rescued orphaned pre‐ and post‐synaptic components in mSOD1 larvae. (A‐I) Representative images of single ventral root projection double‐labeled for SV2 (presynaptic marker, A, D, G), BTX (postsynaptic marker, B, E, H), and colocalization of α‐SV2 and α‐BTX (merged, C, F, I), in WT (A–C), mSOD1 control (D‐F) and mSOD1 treated with 100 µmol/L Cipro/ 1 µmol/L Celecox (G‐I). (Scale bar = 30 µm, scale bar in insets = 15 µm). (J) Quantification of the number of synapses formed, quantified as the number of colocalized SV2 and BTX puncta. (K) Quantification of orphaned presynaptic SV2 puncta over total number of SV2 puncta. (L) Quantification of orphaned postsynaptic α‐BTX staining over total number of α‐BTX puncta. (ns = non significant; *P < 0.05; **P < 0.01; ***P < 0.001; one‐way ANOVA, Tukey post hoc test, *n* = 18 for WT and Cipro/Celecox treated mSOD1 fish and *n* = 36 for control mSOD1 fish).

To test whether the reduced axonopathy seen following Cipro/Celecox treatment is associated with recovery of NMJ structures, pre‐ and postsynaptic structural components were examined by performing whole mount immunohistochemistry of the NMJ (Fig. 4, segments 10–12). Synaptic vesicle 2 (SV2) was used to assess presynaptic integrity while α‐bungarotoxin (α‐BTX) was used to visualize postsynaptic acetylcholine receptor clusters.

Colocalization analysis of SV2 and BTX staining revealed that mSOD1 larvae displayed a reduction in the number of intact synapses at the NMJ compared to WT larvae (*P* < 0.001; Fig. 4A‐C *vs* D‐F, J). Furthermore, mSOD1 larvae had higher proportions of orphaned SV2 puncta (absence of colocalization of α‐BTX) over total SV2 puncta when compared to WT larvae (*P* < 0.001; Fig. 4K), and higher proportion of orphaned α‐BTX receptor staining (absence of colocalization of SV2) over total α‐BTX receptor staining when compared to the WT group (*P* < 0.001; Fig. 4L).

In line with our MN morphological findings, mSOD1 larvae treated with 100µM Cipro/ 1µM Celecox, exhibited a higher number of intact organized synapses, with reduced proportion of orphaned pre‐ and post‐synaptic puncta, resembling WT larvae (*P* < 0.001; Fig. 4G‐I, J‐L).

### Cipro/Celecox preserved the ramified morphology of microglia cells

As neuroinflammation is one of the primary mechanisms underlying the pathophysiology of ALS, we set out to assess the immunological environment induced by Cipro/Celecox in the brain of mSOD1 zebrafish model. To that end, a transgenic line expressing fluorescent microglia cells Tg(Apo‐E:GFP) was used and crossed with WT or mSOD1 lines. The Apo‐E:GFP transgenic larvae express a membrane‐bound GFP marker under the control of the apolipoprotein‐E locus, a previously described marker for zebrafish microglia.^34^

Three‐dimensionally reconstructed ApoE^+^ microglia in the tectum (midbrain) of mSOD1 larvae were morphometrically analyzed. The optic tectum projects through the reticular formation and interacts with MNs in the brain stem.^38^

**Figure 5:**
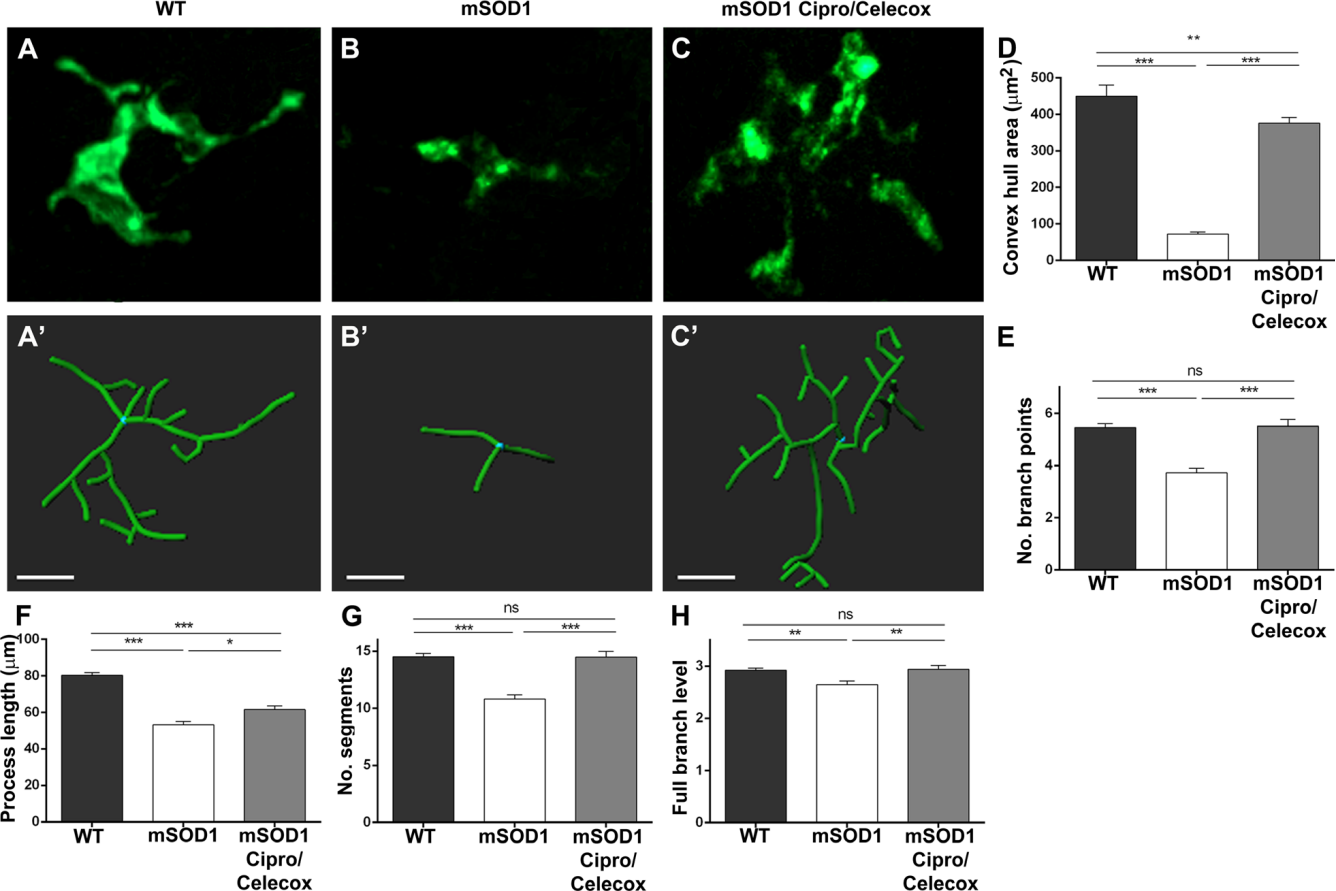
Cipro/Celecox preserved the ramified morphology of surveillant microglia. Morphological analysis of individual microglia in the tectum of zebrafish larvae. (A–C) 3D reconstructed Apotome z‐stack images of branching Apo‐E:GFP microglial cells. (A'–C') The backbone (colored processes) of microglia cells traced with the Filaments analysis of Imaris software. (A, A') WT. (B, B') mSOD1 control treated with vehicle solvent. (C, C') mSOD1 treated with 100 µmol/L Cipro/ 1 µmol/L Celecox. (Scale bar = 10 µm). (D–H) Morphometric analysis measurements of individual microglia in WT, mSOD1 control and mSOD1 treated with Cipro/Celecox. Graphs indicate (D) convex hull area, (E) number of branch points, (F) process length, (G) number of segments and (H) full branch level. (ns = non significant; *P < 0.05; **P < 0.01; ***P < 0.001; one‐way ANOVA, Tukey post hoc test, *n* = 30 for WT fish *n* = 20 for control mSOD1, *n* = 17 for Cipro/Celecox treated mSOD1 fish).

Cell complexity analysis showed a typical ramified morphology of surveillant cells in WT brains as evident by measures of convex hull area, number of branching points, process length, number of segments and branch level (Fig. 5A‐A', D‐H).

Similar quantitative analysis of mSOD1 microglial morphology compared to WT revealed lower spatial microglial coverage (*P* < 0.001), fewer branching points (*P* < 0.001), reduced total branch length (*P* < 0.001), fewer branches (*P* < 0.001), and reduced branch level (*P* < 0.01) (Fig. 5B‐B', D‐H). All parameters of morphometric analysis showed reduction in the complexity of cell morphology, exhibiting reactive phenotype with simpler and shorter processes (Fig. 5D‐H).

Compared to their nontreated siblings, mSOD1 larvae treated with 100µM Cipro/ 1µM Celecox demonstrated higher spatial microglial coverage (*P* < 0.001), more branching points (*P* < 0.001), increased total branch length (*P* < 0.05), more branches (*P* < 0.001) and increased branch level (*P* < 0.01) (Fig. 5C‐C', D‐H), resembling WT microglial phenotype.

### Cipro/Celecox reduced locomotor deficits in zebrafish expressing mutant human *TARDBP*

To further assess whether treatment with Cipro/Celecox may prove beneficial in other ALS models, we used a zebrafish model transiently expressing the *TARDBP* G348C ALS variant.^39^ This model displays hallmarks associated with ALS, including impairment of NMJs and reduced motor performance.^39^

**Figure 6:**
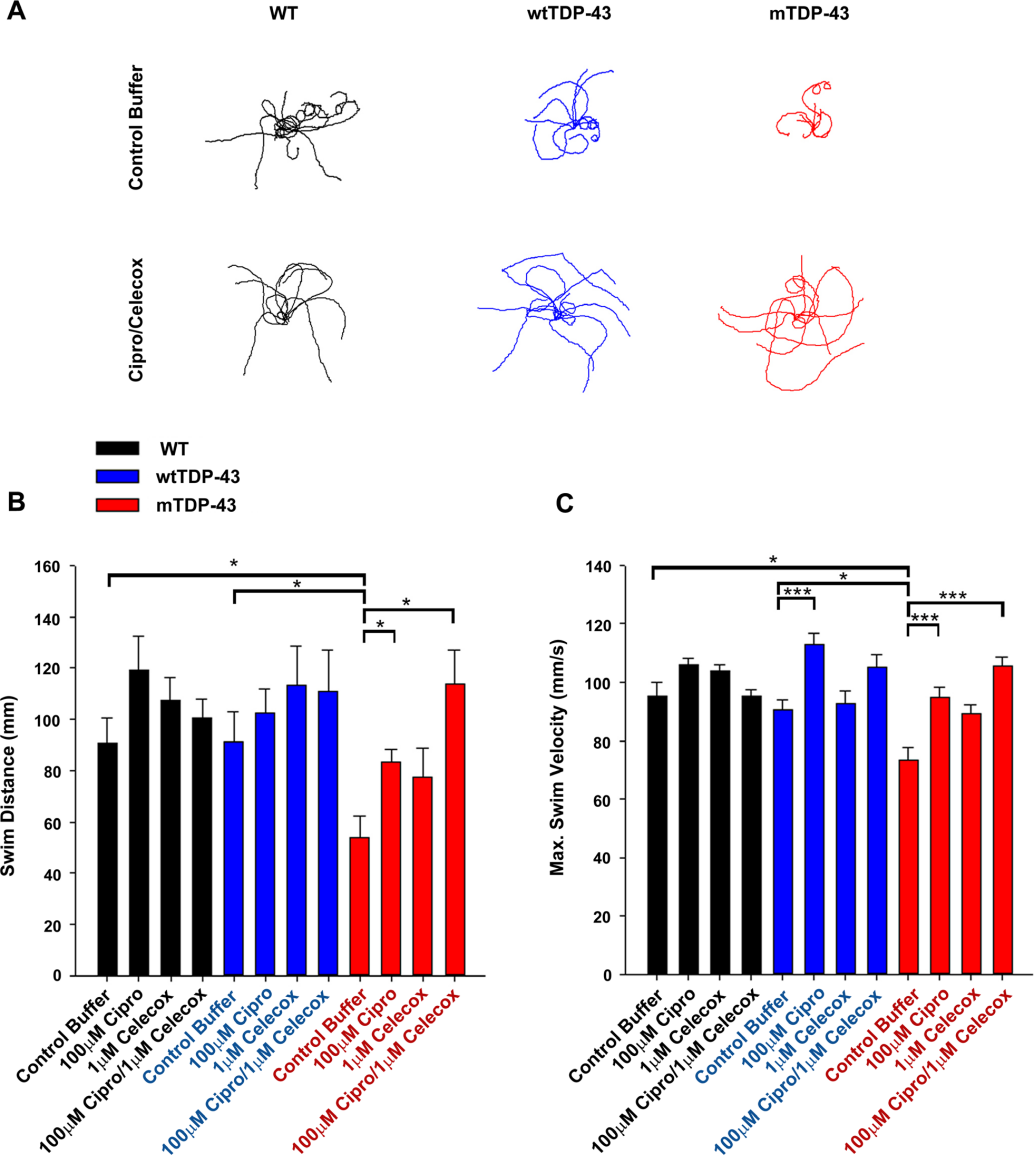
Cipro/Celecox reduced locomotor deficits in zebrafish expressing TDP‐43 mutant. (A) Ten representative traces of touch‐evoked motion paths are superimposed for each genetic group (WT, wtTDP‐43 and mTDP‐43) treated with vehicle or 100 µmol/L Cipro/ 1 µmol/L Celecox combination. (B) Touch‐evoked larval swim distances and (C) maximum swim velocities were measured and calculated following treatment with 1 µmol/L celecoxib, 100 µmol/L ciprofloxacin or 100 µmol/L Cipro/ 1 µmol/L Celecox in all genetic groups. (ns = non significant; *P < 0.05; ***P < 0.001; Kruskal‐Wallis One Way Analysis of Variance on Ranks, Dunn's post hoc test or one‐way ANOVA, Holm‐Sidak multiple comparison test, *n* = 19–28 for WT, *n* = 12–22 for wtTDP‐43, n = 20–23 for mTDP‐43).

Three genetic groups were used: noninjected WT larvae, larvae injected with human WT *TARDBP* mRNA (wtTDP‐43), and larvae injected with human mutant *TARDBP* mRNA (mTDP‐43). Within each genetic group larvae were subdivided into four drug treatment groups: control buffer, 1μM Celecoxib, 100μM Ciprofloxacin, and 100µM Cipro/ 1µM Celecox combination. Efficacy was evaluated by examining larval locomotor function, evoked following a touch to the tail, which reliably initiates burst swimming behavior. As transcripts were expressed transiently, locomotor assays were conducted at 54–56 hpf, when an active response to touch is already established. Swim distance and maximal swim velocity were measured for individual larva (Fig. 6).

Larvae injected with mTDP‐43 displayed evoked touch responses that were significantly shorter in swim distance and reduced in maximal swim velocity when compared to WT larvae or wtTDP‐43‐injected larvae (*P* < 0.05; Fig. 6A‐C).

No differences were found in locomotor responses between vehicle‐treated (control), WT larvae and WT larvae treated with any of the drugs alone or their combination (Fig. 6B, C) and in swim distance in all treated and nontreated wtTDP‐43 larvae (Fig. 6B). Ciprofloxacin increased maximal swim velocity of wtTDP‐43 larvae (*P* < 0.001; Fig. 6C). It is possible that a slight toxic effect that arose as a result of wtTDP‐43 expression was protected by application of Ciprofloxacin.

Examining the mTDP‐43 larvae, Ciprofloxacin induced a significant increase in the distance these larvae swam (*P* < 0.05) and in their maximal swim velocity (*P* < 0.001) (Fig. 6B, C). When Ciprofloxacin was combined with a low dose of Celecoxib, this result was significantly enhanced. Cipro/Celecox‐treated mTDP‐43 larvae showed a significant increase of 110% (*P* < 0.05) in swim distance and 43.8% (*P* < 0.001) in maximal swim velocity compared to control mTDP‐43 larvae (Fig. 6A–C).

These results indicate a robust effect of Cipro/Celecox combination on preserving motor function in zebrafish larvae expressing mutant TDP‐43 (G348C) variant.

## DISCUSSION

Despite extensive efforts that are being made to understand mechanisms and develop drugs for ALS, treatment possibilities remain limited. Here, we utilize two well‐characterized genetic ALS zebrafish models expressing mutant forms of *SOD1* and *TARDBP* to identify a new neuroprotective role for Cipro/Celecox drug combination, suggesting that it may serve as an effective treatment in ALS.

Both zebrafish models, SOD1 G93R and TDP‐43 G348C, recapitulate the hallmarks of ALS, including locomotor impairments, muscular atrophy, MN degeneration, and NMJ loss.^24^, ^25^ Treatment with Ciprofloxacin, combined with a low dose of Celecoxib, caused significant enhancement of locomotor activity in both models, implying that Cipro/Celecox combination specifically and efficiently targets key mechanisms underlying ALS, with the overall outcome of improving motor function.

Ciprofloxacin, a fluoroquinolone antibiotic, is commonly used and has proven its safety following extensive use around the world. Moreover, it can cross the blood–brain barrier (BBB) and has a substantial RNAi‐enhancing activity.^11^ The underlying rationale for using Ciprofloxacin in ALS treatment was that microRNAs are dysregulated in multiple forms of ALS^40^ and improving microRNA regulation was beneficial in two independent ALS mouse models.^41^ In our study, both mSOD1 and mTDP‐43 models treated with Ciprofloxacin alone, displayed improved locomotor activity parameters. As dysregulated microRNAs were found in immune and MN cells of ALS models and patients,^6^, ^7^ the use of Ciprofloxacin can potentially lead to better controlling multiple targets such as neuroinflammation, synaptic formation and stability and neuronal activity.

ALS models and patients show increased levels of COX2 and PGE2.^12^, ^13^, ^14^ Inhibitors of COX2 have been shown to markedly reduce astrocytic glutamate release.^42^ Furthermore, COX2 inhibitors interrupt inflammatory processes.^18^ Celecoxib, a specific COX2 inhibitor, markedly inhibited production of PGE2 in the spinal cords and prolonged survival in the SOD1 G93A mouse model by 25–30%.^19^ However, treatment of ALS patients with 800 mg/day Celecoxib did not result in a beneficial effect in any of the disease parameters measured.^20^ It is possible that Celecoxib in high doses loses its antiinflammatory effect, since in these conditions it can evoke NF‐κB and COX2 activation and induce the transcription of NF‐κB‐dependent genes such as *TNF*.^43^ Furthermore, in these doses, its pharmacokinetic profile may not be linear, affecting the drug's activity.^15^ Nevertheless, Celecoxib itself, at a vast concentrations range, did not significantly enhance locomotor activity in our models, suggesting that its mechanism of action is not sufficient to improve disease parameters.

Remarkably, our data showed a synergistic effect between Celecoxib and Ciprofloxacin in the control and regulation of pathological pathways involved in both ALS zebrafish models. The Cipro/Celecox synergism may potentially control and regulate pathological pathways involved in ALS. Low doses of Celecoxib were shown to play a synergistic role with Ciprofloxacin in regulating OS and inflammation in murine brain abscesses.^21^ Studies have shown that Ciprofloxacin and other fluoroquinolone antibiotics are substrates of MDR1^23^ and Celecoxib can inhibit MDR1, leading to Ciprofloxacin increase inside cells.^22^ Interestingly, SOD1 G93A mouse shows selective increase of ATP‐binding drug efflux transporters at the blood‐spinal cord barrier which suggests induced pharmaco‐resistance also in ALS.^44^ Notably, cerebrospinal fluid concentrations of Ciprofloxacin were increased from 70% to 100% by coadministration of COX inhibitors.^45^ Support for such effect was obtained in our study when comparing mSOD1 locomotor activity to those of the mTDP‐43 model. mSOD1 larvae were treated with a single or combined drugs once the BBB was fully developed.^46^ In this experiment, a clear synergistic effect was evident between the drugs. However, due to constraints of injected‐mRNA stability, mTDP‐43 larval experiments were conducted earlier, before full BBB closure. In this set of experiments, additive effect was evident rather than synergistic, suggesting that Celecoxib may play a role in Ciprofloxacin penetrance to the CNS. Future studies may reveal the exact targets and mechanism of the synergism between Ciprofloxacin and Celecoxib and their relevance to ALS.

mSOD1 larvae exhibited a substantial recovery of MN morphology and NMJ structure, suggesting a potential neuroprotective role for Cipro/Celecox treatment. ALS zebrafish models mutated in distinct ALS‐associated genes, exhibit a phenotype consisting of disorganized, excessively branched MN axons.^25^, ^47^, ^48^, ^49^, ^50^, ^51^ These early phenotypes are in accordance with studies showing that before any loss of spinal cord MNs in the SOD1 G93A mouse, there is a dynamic process of new axonal sprouting continuously denervating and reinnervating nearby NMJs (some of which may not be functional).^52^, ^53^ Evidence from an autopsy of an ALS patient indicated that denervation and reinnervation of NMJs might occur mid disease course.^53^ Interestingly, mSOD1 fish showed a remarkable recovery of MN morphology following Cipro/Celecox treatment, recapitulating near normal axonal morphology. This reduction in axonal sprouting, may suggest that motor‐units are not fully dismantled following Cipro/Celecox treatment and that axonal sprouting to reinnervate orphaned NMJs may not be required. Following treatment with Cipro/Celecox, mSOD1 larvae exhibited a higher number of intact organized synapses, with reduced proportion of orphaned pre‐ and post‐synaptic puncta, suggesting protection against worsening of impaired NMJ transmission. Whether Cipro/Celecox combination reduces abnormal motor axonal sprouting due to the stabilization of NMJs or NMJ stabilized structures are a consequence of reduction in MN pathology remains to be elucidated. However, both mechanisms support the development of Cipro/Celecox treatment for clinical trials in ALS patients. Neural improvement cannot explain the complete phenotype we see. This suggests that part of the phenotype may result from improvement in muscular components, and will need to be further resolved.

Our findings suggest that Cipro/Celecox combination could reverse reactive microglia to surveillant cells, possibly following reduction of signals manifested as a result of reduced axonopathy. In a variety of neurodegenerative diseases including ALS, “degeneration‐ or disease‐associated microglia” alter their transcriptional profile, morphology and function.^54^ Evidence of microglial activation in the cortex of ALS patients has been obtained from postmortem studies^55^ and *in vivo* in ALS patients using glial activation biomarkers imaging.^56^ In agreement with these findings and with microglial morphometrical analyses conducted in SOD1 G93A mice,^57^ microglia cells in mSOD1 larvae exhibit activated phenotype with impaired process complexity. When treated with Cipro/Celecox combination, microglia in the brains of mSOD1 larvae exhibited ramified morphology, with higher coverage of the parenchyma, resembling surveillant cells as in healthy larvae. Further studies are needed to clarify whether this phenotype is caused by reduced signals from MN or alteration in microRNA regulation.

Though the effect we see may be relevant for early development of ALS phenotypes, many papers show that models presenting a series of ALS‐like early phenotypes can be used as a basis for drug screening in its larval stages,^26^, ^50^, ^58^, ^59^, ^60^, ^61^ and as a whole, many drugs at distinct phases of clinical trials in adult humans originated in successful drug‐screen in larvae zebrafish stages.^32^

Changes in the expression of specific miRNAs, neuroinflammation and excessive production of reactive oxygen species have been implicated also in the pathogenesis of other neurodegenerative diseases, such as Parkinson’s disease, Alzheimer’s disease, and Huntington disease. Therefore, it may be interesting to test the neuroprotective role of Cipro/Celecox combination for the treatment of other indications as well. Nevertheless, when translating this combined treatment to human patients, two aspects will need to be considered: long‐term antibiotics resistance and the microbiome composition of ALS patients.

The overall results presented here point to a substantial neuroprotective effect of Cipro/Celecox combination in two ALS zebrafish models and suggest it could serve as an effective treatment to alter disease progression.

## Author contributions

A.P., J.M.S., G.A.B.A., and N.R.B. contributed to the study concept and design. H.G., A.M., V.P.L., R.R., R.T.P., G.A.B.A., and N.R.B. contributed to data acquisition and analysis. H.G., A.M., V.P.L., A.P., G.A.B.A., and N.R.B. contributed to drafting the manuscript and figures.

## Conflicts of interest

G.A.B.A. and N.R.B. received research funding from NST. For a portion of the project duration, J.M.S., R.T.P. and N.R.B. report personal consulting fees from NST. A.P. is an employee of NST. NST owns patent rights to Cipro/Celecox combination that was used in this study. The other authors declare no conflicts of interest.

## Supporting information


**Figure S1** **.** Effect of dark‐light transition on locomotion in WT vs. mSOD1 larvae. A 10‐min period of darkness was followed by 10‐min light phase. Black and white bars at the bottom signify dark and light conditions, respectively. Data are presented as mean ± S.E.M. distance moved of WT and mSOD1 larvae (n = 72/line). Based on averaged total activity of 1‐min bin for 10‐min period. Our analysis indicated a significant difference between WT and mSOD1 locomotor activity in each condition (dark p < 0.005; light p < 0.0001). Data were analyzed using linear mixed effects model (with genetic background as a fixed effect and a random intercept for each plate). Time “10” here is time “0” in the main figures (light stimuli).
**Figure S2** **.** Increase in Ciprofloxacin dosing did not cause further locomotor improvement. mSOD1 larvae were treated with vehicle (0.1% DMSO; Ct), 200µM or 500µM Ciprofloxacin, and were then subjected to dark/ light transition. The distance they swam per time bin of 1 min following light stimuli was measured and averaged. (*p < 0.05; **p < 0. 01; linear mixed model, Tukey post hoc test, n = 96 for each treatment group).
**Figure S3** **.** Low Celecoxib doses had no effect on mSOD1 activity. mSOD1 larvae were treated with vehicle (0.1% DMSO; Ct), 0.1µM or 0.5µM Celecoxib, and were then subjected to dark/ light transition. The distance they swam per time bin of 1 min following light stimuli was measured and averaged. (ns = non significant; linear mixed model, Tukey post hoc test, n = 96 for each treatment group).
**Table S1** **.** Locomotor activity of treated mSOD1 larvae during the dark phase supports the synergistic effect of the drugs. Data are presented as mean ± S.E.M. distance moved (in mm) of 96‐123 larvae. Based on activity summed within each 1‐min period and averaged for the 10‐min dark period, linear mixed effects model (with treatment as a fixed effect and a random intercept for each plate) indicated a significant difference of locomotor activity in the combination‐treated mSOD1 larvae (*P* < 0.05). WT larvae treated with vehicle (WT Ct) or combination of the drugs did not show a significant difference of locomotor activity.
