# Supplementary material for: Efficacy of Ciprofloxacin/Celecoxib combination in zebrafish models of amyotrophic lateral sclerosis
Source: Ann Clin Transl Neurol. 2020 Sep 11;7(10):1883–97. doi: 10.1002/acn3.51174 (PMC7545590; doi:10.1002/acn3.51174)
Supplement: Supplementary file 1 — Figure S1 . Effect of dark‐light transition on locomotion in WT vs. mSOD1 larvae. A 10‐min period of darkness was followed by 10‐min light phase. Black and white bars at the bottom signify dark and light conditions, respectively. Data are presented as mean ± S.E.M. distance moved of WT and mSOD1 larvae (n = 72/line). Based on averaged total activity of 1‐min bin for 10‐min period. Our analysis indicated a significant difference between WT and mSOD1 locomotor activity in each condition (dark p < 0.005; light p < 0.0001). Data were analyzed using linear mixed effects model (with genetic background as a fixed effect and a random intercept for each plate). Time “10” here is time “0” in the main figures (light stimuli). Figure S2 . Increase in Ciprofloxacin dosing did not cause further locomotor improvement. mSOD1 larvae were treated with vehicle (0.1% DMSO; Ct), 200µM or 500µM Ciprofloxacin, and were then subjected to dark/ light transition. The distance they swam per time bin of 1 min following light stimuli was measured and averaged. (*p < 0.05; **p < 0. 01; linear mixed model, Tukey post hoc test, n = 96 for each treatment group). Figure S3 . Low Celecoxib doses had no effect on mSOD1 activity. mSOD1 larvae were treated with vehicle (0.1% DMSO; Ct), 0.1µM or 0.5µM Celecoxib, and were then subjected to dark/ light transition. The distance they swam per time bin of 1 min following light stimuli was measured and averaged. (ns = non significant; linear mixed model, Tukey post hoc test, n = 96 for each treatment group). Table S1 . Locomotor activity of treated mSOD1 larvae during the dark phase supports the synergistic effect of the drugs. Data are presented as mean ± S.E.M. distance moved (in mm) of 96‐123 larvae. Based on activity summed within each 1‐min period and averaged for the 10‐min dark period, linear mixed effects model (with treatment as a fixed effect and a random intercept for each plate) indicated a significant difference of locomotor activity in the combin [file ACN3-7-1883-s001.docx]

**Supplementary Material**

**
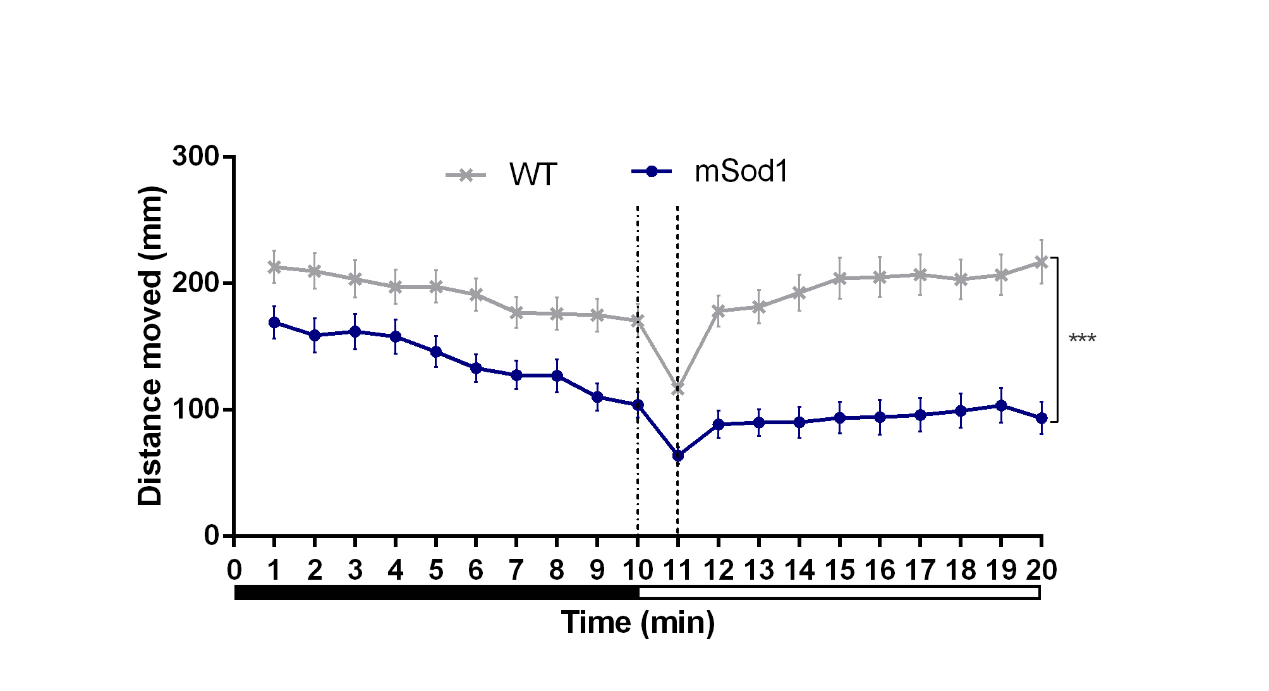
Supplementary Figure 1. Effect of dark- light transition on locomotion in WT vs. mSod1 larvae.** A 10-min period of darkness was followed by 10-min light phase. Black and white bars at the bottom signify dark and light conditions, respectively. Data are presented as mean ± S.E.M. distance moved of WT and mSod1 larvae (n=72/line). Based on averaged total activity of 1-min bin for 10-min period. Our analysis indicated a significant difference between WT and mSod1 locomotor activity in each condition (dark p<0.005; light p< 0.0001). Data was analyzed using linear mixed effects model (with genetic background as a fixed effect and a random intercept for each plate). Time “10” here is time “0” in the main figures (light stimuli).

**
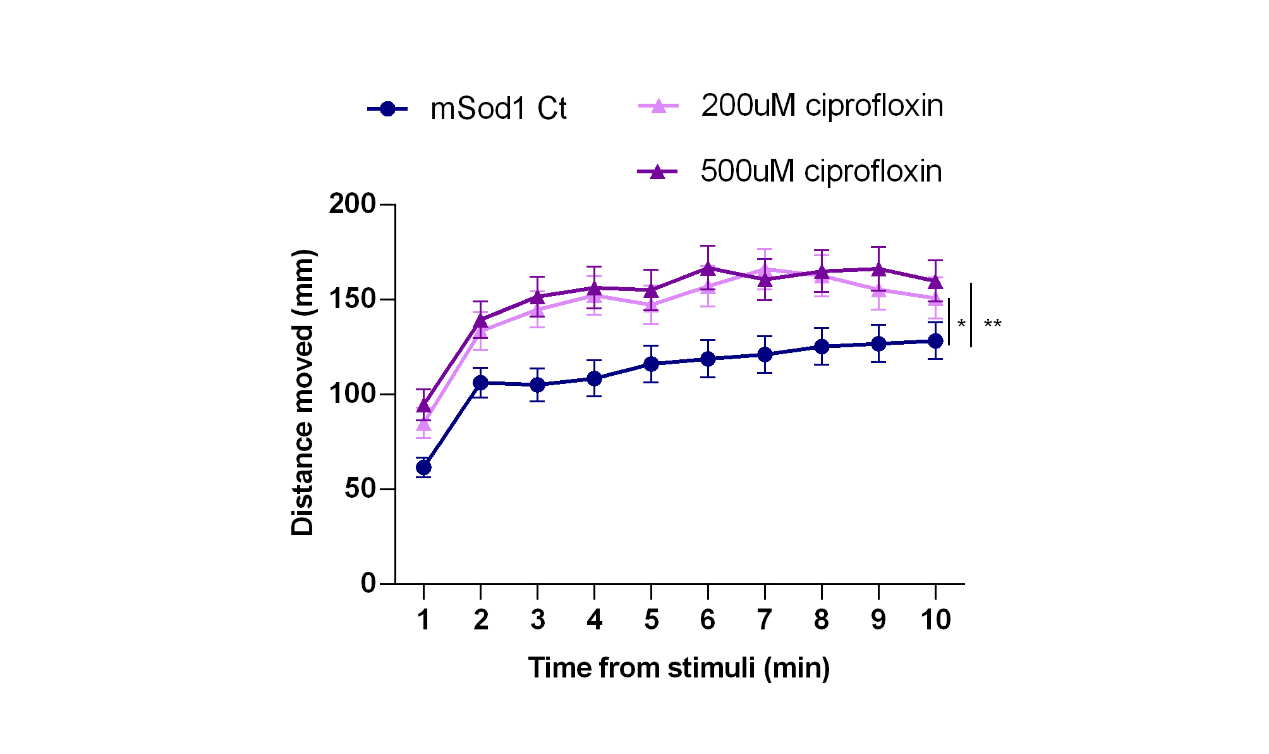
**

**Supplementary Figure 2.** **Increase in Ciprofloxacin dosing did not cause further locomotor improvement.** mSOD1 larvae were treated with vehicle (0.1% DMSO; Ct), 200μM or 500μM Ciprofloxacin, and were then subjected to dark/ light transition. The distance they swam per time bin of 1 min following light stimuli was measured and averaged. (*p< 0.05; **p< 0. 01; linear mixed model, Tukey post-hoc test, n=96 for each treatment group).

**
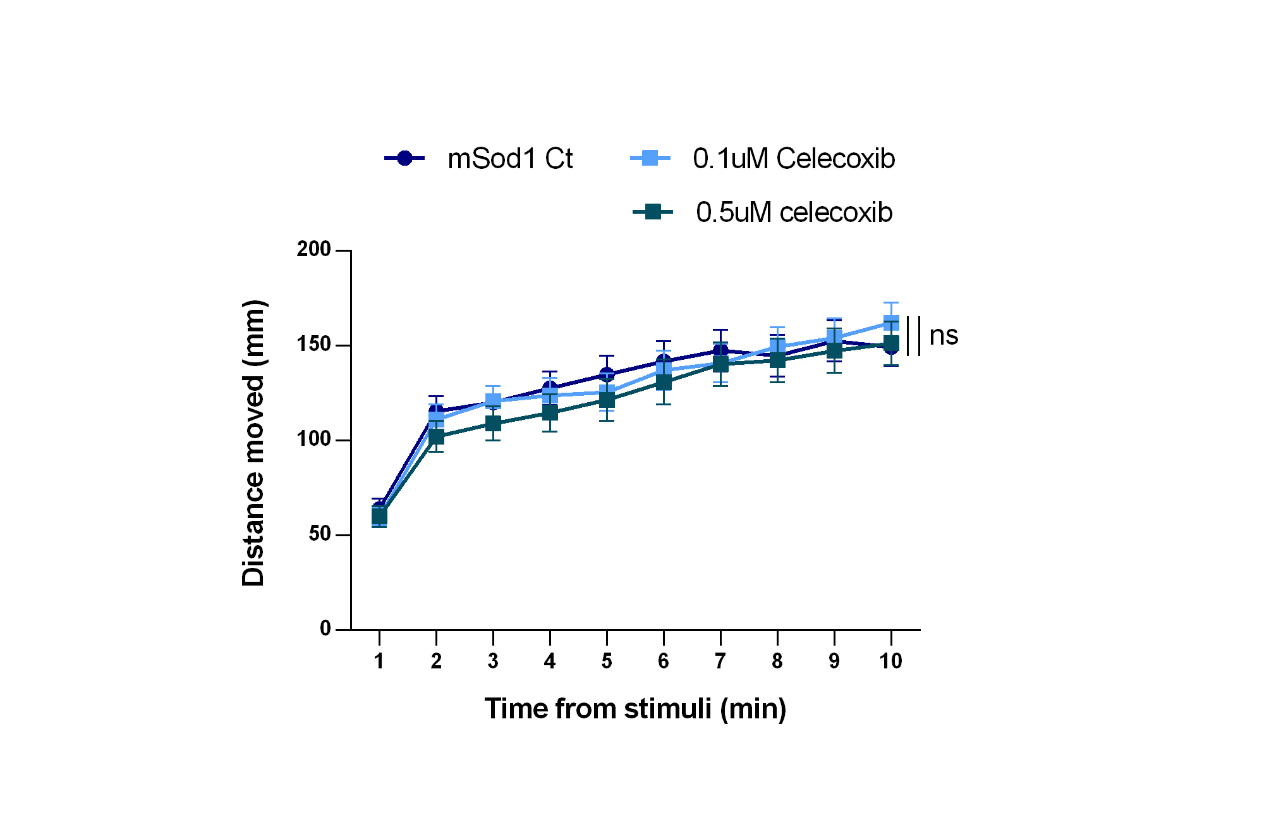
**

**Supplementary Figure 3.** **Low Celecoxib doses had no effect on mSOD1 activity.** mSOD1 larvae were treated with vehicle (0.1% DMSO; Ct), 0.1μM or 0.5μM Celecoxib, and were then subjected to dark/ light transition. The distance they swam per time bin of 1 min following light stimuli was measured and averaged. (ns = non significant; linear mixed model, Tukey post-hoc test, n=96 for each treatment group).

**
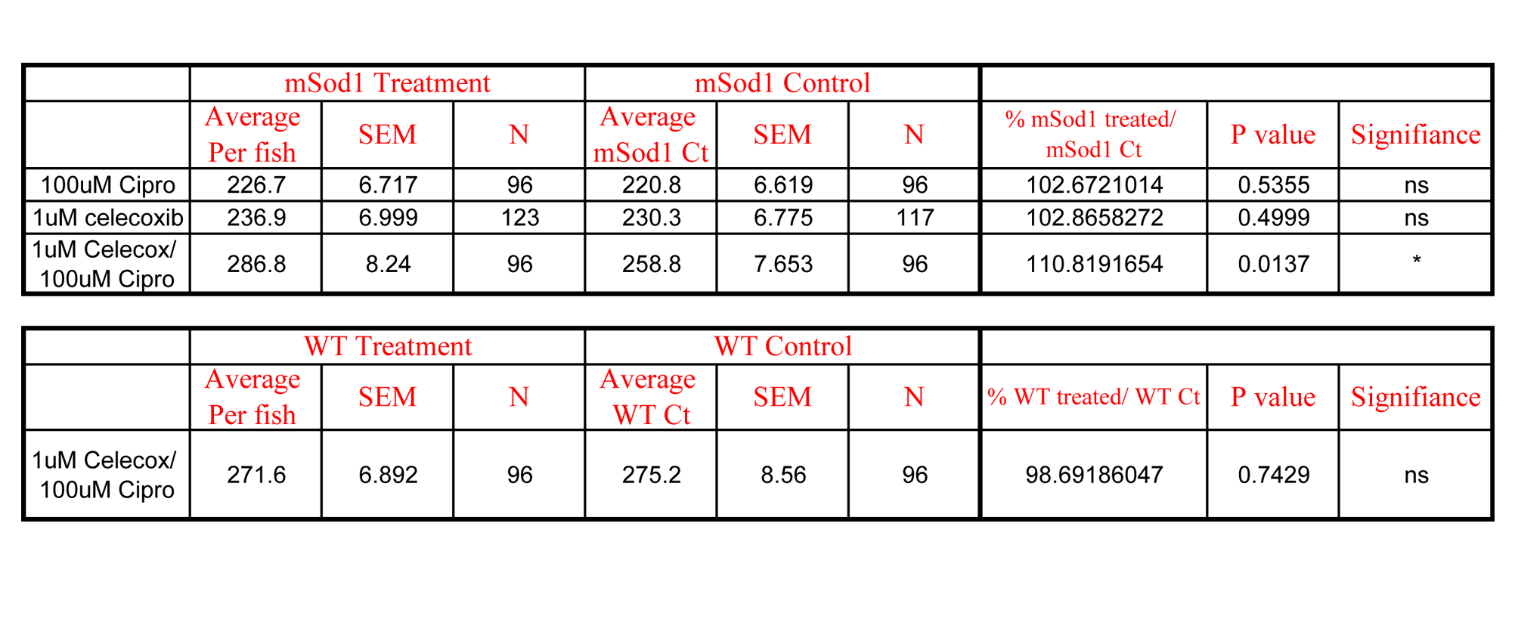
**

**Supplementary Table 1.** **Locomotor activity of treated mSod1 larvae during the dark phase supports the synergistic effect of the drugs.** Data are presented as mean ± S.E.M. distance moved (in mm) of 96-123 larvae. Based on activity summed within each 1-min period and averaged for the 10-min dark period, linear mixed effects model (with treatment as a fixed effect and a random intercept for each plate) indicated a significant difference of locomotor activity in the combination- treated mSod1 larvae (p<0.05). WT larvae treated with vehicle (WT Ct) or combination of the drugs did not show a significant difference of locomotor activity.
